# Progress in 3D Printing Applications for the Management of Orbital Disorders: A Systematic Review

**DOI:** 10.3390/bioengineering11121238

**Published:** 2024-12-07

**Authors:** Luca Michelutti, Alessandro Tel, Massimo Robiony, Salvatore Sembronio, Riccardo Nocini, Edoardo Agosti, Tamara Ius, Caterina Gagliano, Marco Zeppieri

**Affiliations:** 1 Clinic of Maxillofacial Surgery, Head-Neck and NeuroScience Department, University Hospital of Udine, 33100 Udine, Italy; micheluttiluca.uniud@gmail.com (L.M.); alessandro.tel@icloud.com (A.T.);; 2 Section of Ear Nose and throat (ENT), Department of Medical and Surgical Sciences, Dentistry, Gynecology and Pediatrics, University of Verona, 37124 Verona, Italy; 3 Division of Neurosurgery, Department of Medical and Surgical Specialties, Radiological Sciences and Public Health, University of Brescia, Piazza Spedali Civili 1, 25123 Brescia, Italy; 4 Neurosurgery Unit, Head-Neck and NeuroScience Department, University Hospital of Udine, p.le S. Maria della Misericordia 15, 33100 Udine, Italy; 5 Department of Medicine and Surgery, University of Enna “Kore”, Piazza dell’Università, 94100 Enna, Italy; 6 Mediterranean Foundation “G.B. Morgagni”, 95125 Catania, Italy; 7 Department of Ophthalmology, University Hospital of Udine, 33100 Udine, Italy

**Keywords:** orbital diseases, surgical planning, personalized treatment, biocompatibility

## Abstract

**Introduction**: 3D printing technology has gained considerable interest in the domain of orbital illnesses owing to its capacity to transform diagnosis, surgery planning, and treatment. This systematic review seeks to deliver a thorough examination of the contemporary applications of 3D printing in the treatment of ocular problems, encompassing tumors, injuries, and congenital defects. This systematic review of recent studies has examined the application of patient-specific 3D-printed models for preoperative planning, personalized implants, and prosthetics. **Methods**: This systematic review was conducted according to the PRISMA guidelines. The PICOS is “What are the current advances and applications of 3D printing for the management of orbital pathology?” The databases analyzed for the research phase are MEDLINE, Embase, Cochrane Central Register of Controlled Trials (CENTRAL), ClinicalTrials.gov, ScienceDirect, Scopus, CINAHL, and Web of Science. **Results**: Out of 314 studies found in the literature, only 12 met the inclusion and exclusion criteria. From the included studies, it is evident that 3D printing can be a useful technology for the management of trauma and oncological pathologies of the orbital region. **Discussion**: 3D printing proves to be very useful mainly for the purpose of improving the preoperative planning of a surgical procedure, allowing for better preparation by the surgical team and a reduction in operative time and complications. **Conclusions**: 3D printing has proven to be an outstanding tool in the management of orbit pathology. Comparing the advantages and disadvantages of such technology, the former far outweigh the latter.

## 1. Introduction

3D printing technology has had a strong growth phase in the past decade. Thanks to cost reductions, technological innovation, and research investments, it has spread to various branches of medicine, cardiac surgery, reconstructive plastic surgery, orthopedics, and especially maxillofacial surgery [1,2,3]. The use of 3D printing has proven to be an excellent tool for the surgeon to improve the quality and precision of surgical procedures that require great accuracy. 3D printing allows for the ability to create customized medical devices, resection guides, prostheses, and implants, improving the outcome of surgery in both functional and aesthetic terms [4,5]. The findings indicate that 3D printing boosts surgical accuracy, decreases operative duration, and improves patient outcomes through tailored treatment methodologies. Furthermore, obstacles including material biocompatibility, elevated costs, and regulatory concerns are addressed. This analysis emphasizes the transformative influence of 3D printing in orbital disease care and identifies avenues for future research and technical progress.

### 1.1. Orbital Diseases

The focus of our study is represented by orbital pathology. Although the orbit is a small anatomical site, it can harbor various diseases. These include inflammatory diseases, such as orbital cellulitis and *Pseudotumor Orbitae*, autoimmune diseases, such as Graves’ ophthalmopathy, traumatic diseases, and oncological diseases [6,7,8].

Fractures of the orbit can be divided into various groups, into lesions of the orbital rim, zygomatic-malar complex (ZMC), nasoorbitoethmoidal (NOE) region, and frontal region. These conditions can present with various symptoms including pain, hematomas, diplopia, enophthalmos or exophthalmos, and reduced ocular mobility [9,10]. Oncological diseases include benign tumors, such as cavernous hemangiomas, dermoid cysts, and pleomorphic adenoma of the lacrimal gland, and malignant tumors, such as orbital lymphoma, rhabdomyosarcoma, and adenoid cystic carcinoma of the lacrimal gland [11,12].

These are conditions that can lead to the appearance of many pre-, intra-, and postoperative complications, such as diplopia, enophthalmos, and exophthalmos, and this also depends in part on the type of approach taken and the difficulty that the surgeon may have during the surgical phase. In fact, according to the study conducted by Govind et al. (2023) [13], trauma involvement of the medial wall of the orbit results in greater surgical difficulty. 3D printing could help reduce the difficulty of surgery by improving surgical planning.

### 1.2. Stages of Making 3D Models

**Figure 1:**
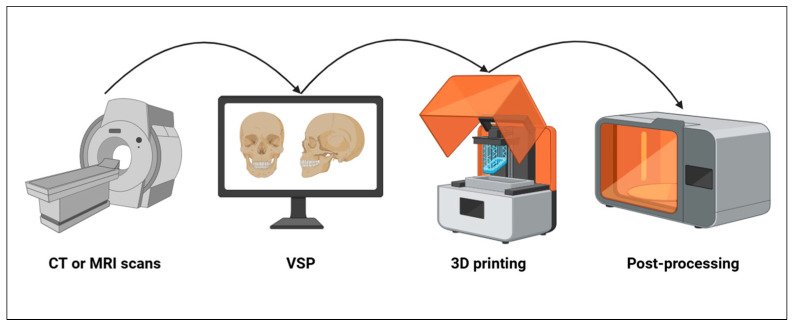
Stages of making the 3D model.

**Figure 2:**
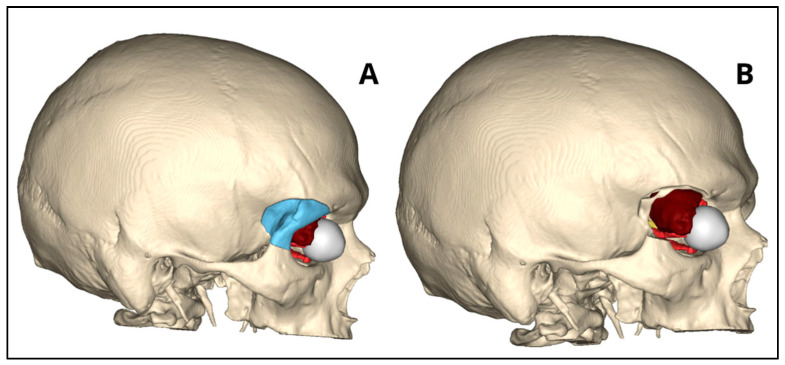
Example of virtual surgical planning (VSP) of a patient with neoplasm of the orbit. (**A**) 3D virtual reconstruction with bone before osteotomy. (**B**) 3D virtual reconstruction after osteotomy with exposure of the tumor lesion.

**Figure 3:**
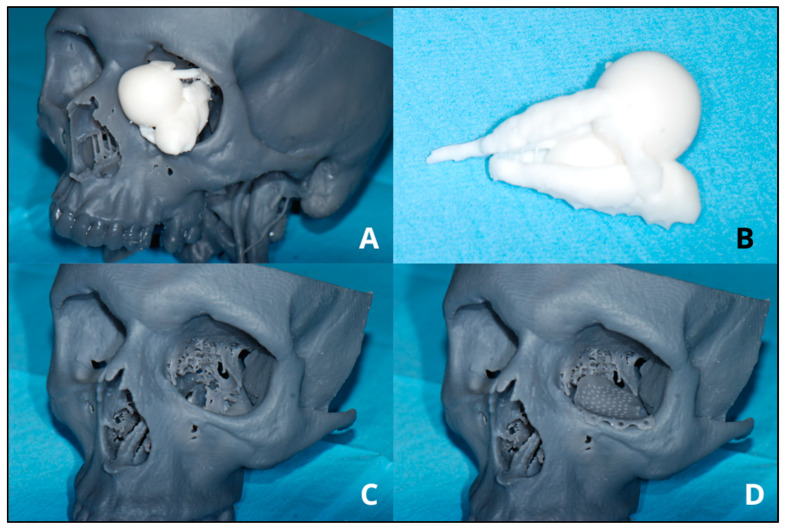
Example of 3D-printed models. (**A**) Eyeball reconstruction with a tumor in the orbital site. (**B**) Eye globe reconstruction, medial and inferior rectus muscle, tumor. (**C**) Reconstruction left orbital floor fracture. (**D**) Orbital floor reconstruction with mesh placement. Mesh is a reproduction created for teaching purposes, not implantable.

**Figure 4:**
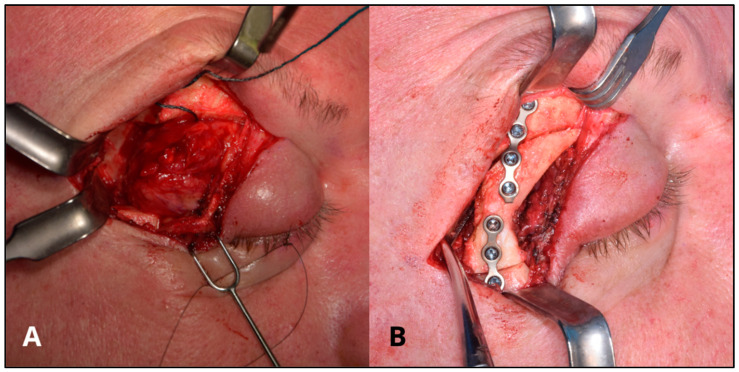
(**A**) Surgical tumor removal surgery. (**B**) Osteosynthesis with plates and screws.

Several steps are required for the creation of a 3D model, Figure 1 briefly shows the steps involved in making a 3D model from CT or MRI scans of the patient. It starts with the acquisition of CT or MR scans of the patients. The production of a CAD (computer-aided design) model is accomplished through CAD software (Mimics innovation suite v.26.0, Materialise NV, Leuven, BE) or by generating a 3D representation from DICOM (Digital Imaging and Communications in Medicine) data obtained from CT or MRI scans. Through various VSP (Virtual Surgical Planning) software (Mimics innovation suite v.26.0, Materialise NV, Leuven, BE), anatomical replicas, surgical guides, or implants can be designed and manufactured based on the specific anatomical features of individual patients. Using specialized software, it is possible to plan the surgery by simulating osteotomies, making the necessary bone movements and corrections (Figure 2). In addition, it is possible to virtually make custom devices necessary to make the surgery more precise and accurate such as splints and surgical templates (Figure 3). Once the virtual 3D model is created, it is stored in a standardized .STL format. The .STL file with the design is sent to the printer, and depending on the object to be printed, specific materials and machine types are selected. After printing the 3D model, post-processing is required, such as polishing, curing, chemical treatment, and the removal of supporting structures. Once the 3D model is made, it is sterilized through various procedures and brought into the room to guide the surgeons during surgery (Figure 4) [14,15].

**Figure 5:**
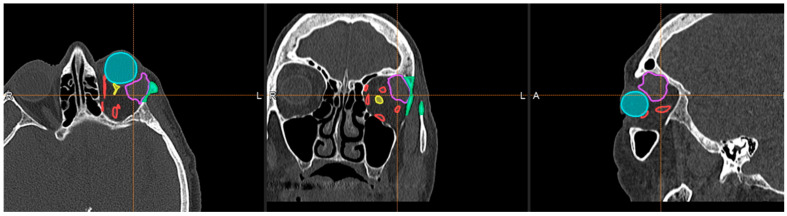
Example of navigation surgery and 3D reconstruction anatomical structures and neoplasia of the orbit. In the image the muscles are red, the optic nerve is yellow, the osteotomy is green, the orbital tumor is violet, and the eyeball is cyan.

In addition, during surgery, it is possible to take advantage of technologies that can provide real-time feedback on the position of instruments and 3D surgical models with respect to anatomical structures, such as surgical navigation. In fact, this other tool can help surgeons and further improve the accuracy and customization of the surgery by adapting to the patient’s specific anatomical features (Figure 5). [16,17]

### 1.3. Types of Printers and Materials Used in 3D Printing

There are different types of printers, each with specific properties and uses. Among the most common are stereolithography (SLA) printers, selective laser sintering (SLS) printers, fused material deposition (FDM) printers, powder bed fusion (PBF) printers, direct metal lase sintering (DMLS) printers, multijet modelling (MJM) printers, and digital light curing (DLP) [18,19,20,21,22]. These machines differ in mainly in the mode of functioning and the type of materials used. The main features are shown in Table 1.

Depending on the 3D model to be printed, specific materials are used based on their properties. The study conducted by Martelli et al., 2016 [23] investigated what the applications of 3D printing are in the surgical field by reporting which materials are most used. Table 2 summarizes some of these materials correlated with the specific printer of use [24,25].

### 1.4. Objective of This Study

There are several studies that have been investigating the application of this technology in the medical field for years, particularly in surgery, from neurosurgery to gynecology to maxillofacial surgery. For example, from the review conducted by Cooke et al. (2023), the use of 3D printing in gynecology appears to be useful in the management of oncologic, pediatric, and urogynecology conditions, particularly in surgical planning, education, and the production of custom devices. 3D printing is proving to be an excellent tool for surgical planning, the creation of patient-specific medical devices, and surgical education [26,27,28].

According to the review conducted by Portnoy et al. (2023), 3D printing is increasingly integrating into the practical reality of surgeons [29]. Our study aims to investigate the application of 3D printing in the field of maxillofacial surgery and particularly in the management of orbital pathologies, analyzing the applications of this technology, the types of printers used, and the materials adopted. We believe that 3D printing can be a useful tool for the surgeon, especially in understanding what type of surgery and surgical approach to implement. In fact, there are several ways to address orbital pathology, especially of a traumatic nature: endoscopic approaches and external surgical approaches such as transconjunctival, subciliary, subtarsal, and transcaruncular. According to the systematic review conducted by Palavalli et al. (2023) [30], the different approaches have different complication rates, especially the transconjunctival approach, followed by subciliary and endoscopic. 3D printing may be able to lower these complication rates by improving the preoperative planning and visualization of the lesion by the surgeon.

## 2. Materials and Methods

This systematic review was conducted according to the Preferred Reporting Items for Systematic Review and Meta-Analyses (PRISMA) statement [31], and it was registered in the PROSPERO database (Internation Prospective Register of Systematic Review; ID number 610656).

The PICOS framework (Table 3) on which this study is based can be summarized as follows: “What are the current advances and applications of 3D-printing for the management of orbital pathologies?”.

### 2.1. Literature Search

The literature search was conducted until 20 October 2024, using the following query: *(3d printing) AND ((orbital disease) OR (orbital disorders) OR (orbital cancer) OR (orbital illness) OR (orbital tumor))*. The medical literature databases consulted were MEDLINE, Embase, Cochrane Central Register of Controlled Trials (CENTRAL), ClinicalTrials.gov, ScienceDirect, Scopus, CINAHL, and Web of Science. The query used on MeSH was *(“Printing, Three-Dimensional”[Mesh]) AND “Orbital Diseases”[Mesh]*. The types of included articles are clinical trials, randomized clinical trials, research articles, original articles, and cohort studies. We decided to also consider case series for the paucity of articles in the literature concerning this topic. The time span considered for this search was 10 years.

The articles found in the medical literature databases were imported into EndNote (Clarivate, Analytics, Philadelphia, PA, USA). The articles screening was conducted by two different and independent investigators (L.M. and A.T.), analyzing the title and the abstract, as reported in the PRISMA flowchart. When necessary, in case of doubt, a third investigator was involved (M.R.).

### 2.2. Inclusion and Exclusion Criteria

We have included only studies dealing with 3D printing applied in the management of orbital pathologies. The included studies are only in English and were conducted only on human species and with adult subjects older than 18 years of age. In addition to randomized clinical trials and cohort studies, case series were also included because of the paucity of clinical trials in the literature. We excluded studies without abstracts and which dealt with other technologies outside of 3D printing and other pathologies not related to the orbital cavity.

### 2.3. Data Collection

From the studies included in this systematic review, we extracted the following data: the type of studies included, the nature of the pathology of the orbit, the number of patients, the type and motive of the application of 3D printing, the type of printer, the material used, and the advantages and disadvantages of this technology in terms of complications, operative time, cost, and functional and aesthetic results. The extracted data were collected in a Microsoft Excel spreadsheet by two independent investigators (L.M and A.T.).

### 2.4. Bias Assessment

For the bias assessment, the Robvis Tool (Robvis tool version number 0.3.0.900) [32] was used by the independent investigators (L.M and A.T.). The types of bias examined are bias arising from the randomization process, bias due to missing outcome data, bias in reported outcome selection, bias due to deviations from the intended interventions, and bias in outcome measurement. We decided to use this tool because it is designed to visualize bias risk assessments and helps to improve the clarity and effectiveness of reporting results.

## 3. Results

**Figure 6:**
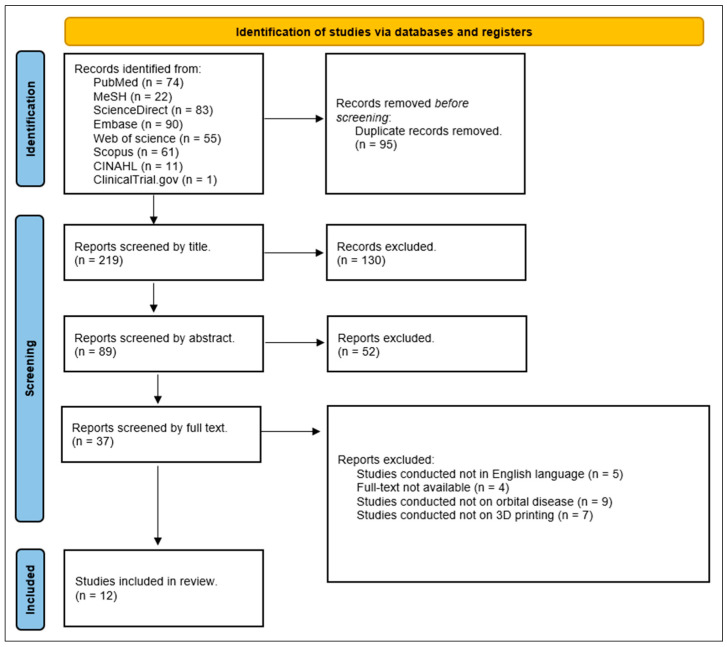
PRISMA flowchart of the systematic review process.

The selection of articles for inclusion followed the PRISMA guidelines, and Figure 6 shows the PRISMA flowchart describing the study selection process. Using a combination of keywords in the various medical databases, the investigators found 314 studies. After reporting all studies found in EndNote, 83 duplicates were identified and removed. The remaining 219 studies were screened first by title, obtaining 89 studies, and later by abstract, excluding 52 studies. The remaining 37 studies were screened by reading the full text, and applying the inclusion and exclusion criteria, 12 studies were included in this review.

**Figure 7:**
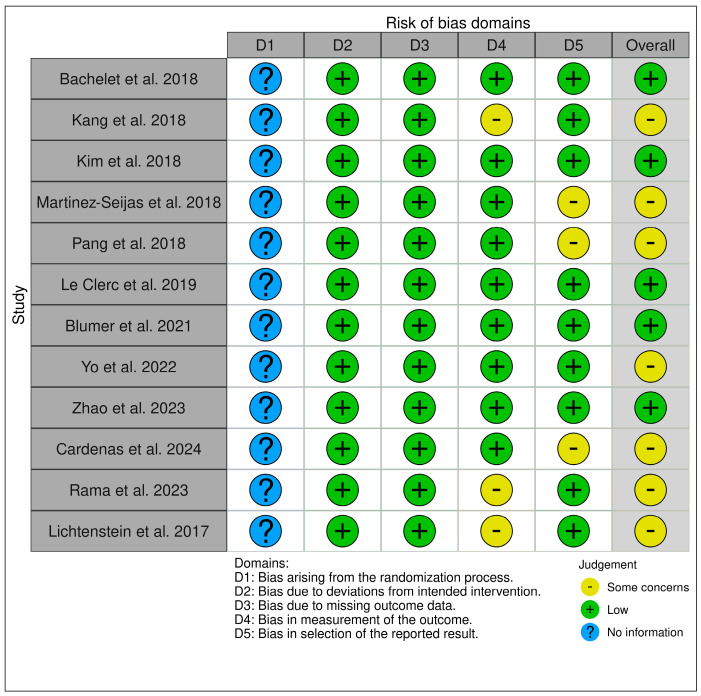
Robvis tool for assessing the risk of bias in the studies included [33,34,35,36,37,38,39,40,41,42,43,44].

The 12 included studies were analyzed, and the risks of bias were assessed through the application of the Robvis Tool [32], as shown in Figure 7. We point out that almost all of the included articles are case series, given the paucity of randomized clinical trials in the literature on the topic of our study. In studies included in this review, which are mainly case series, the bias arising from the randomization process could not be determined. Overall, the other biases prove to be mainly low, with some instead being “some concerns”.

### 3.1. Pathologies of the Orbit Investigated

The pathologies investigated in the included studies are mainly trauma, benign and malignant tumors, and conditions including Basedow’s orbitopathy. Fifty percent of the included studies investigate the application of 3D printing in the management of both unilateral and bilateral fractures, and particularly fractures of the orbital floor and the medial wall. In total, 16.6% of studies focus on the management of tumor pathologies, both benign and malignant, such as dermoid cysts, adenoid cystic carcinomas, cavernous hemangiomas, and adenomas. Additionally, 8.3% deal with dysthyroid optic neuropathy, while another 8.3% focus on the reconstruction of patients with hemifacial microsomia. Finally, two studies apply 3D printing technology without specifying a specific pathology.

### 3.2. Kind of Application of 3D Printing

The main applications of 3D printing used in the included studies are various. In some studies, the direct printing of implants and custom-made prosthetic material, generally made of titanium and other implantable materials, takes place. In other studies, 3D model making is used to recreate bone defects or the fracture to model the plate or mesh to be implanted on them during surgery. Another popular application is the printing of 3D models of surgical templates and other instruments. Finally, 3D printing proves to be a useful tool for the surgical education of trainees and students through the reproduction of traumatic and oncological pathologies of the orbit.

### 3.3. Materials Used

The materials used for making 3D models in the included studies are of various kinds. These include first and foremost titanium, an implantable material par excellence. Other materials used are photopolymerized plastic, wax, porous polyethylene, polymethyl methacrylate (PMMA), acrylonitrile butadiene styrene, plaster, polyetheretherketone (PEEK), polylactic acid plastic filament, and polyamide. Table 4 shows the main features of these materials and their implantability.

Some of the data extracted from the included articles are summarized in Table 5; among them are topic of the study, the number of patients, the type of orbital disease, how 3D printing is applied, and the type of material used.

### 3.4. Advantages and Disadvantages of 3D Printing

3D printing can have several advantages and disadvantages. From the analysis of the included studies, we have elaborated on the following pros and cons of applying 3D printing in the management of traumatological and oncological pathology of the orbit, which are listed in Table 6.

One of the main advantages of 3D printing is to improve preoperative planning, allowing for the better definition of the lesion site in relation to the adjacent anatomical structures. Improving preoperative planning allows the surgeon to be clear about the approach and surgical techniques to be implemented to treat the pathology, thus achieving shorter operative times, reduced intra- and postoperative complications, and better aesthetic and functional outcomes. In addition, this technology proves useful for educational purposes, enabling especially students and young physicians to better understand the disease to be treated.

However, 3D printing does not fully abate the risk of complications and re-intervention, and an additional disadvantage is the timelines required for virtual surgical planning and 3D printing, not to mention all the time required for the post-processing of the printed model, which very often includes washing and curing steps.

## 4. Discussion

This systematic review aims to provide a general overview of what the applications of 3D printing are in the management of pathologies of the orbit. 3D printing technology has had a strong expansion and diffusion in the last 10 years in many fields, especially in the medical field. There are several branches of medicine and especially surgery that adopt this technology for the management of many pathologies, such as reconstructive plastic surgery, dental surgery, orthopedics, cardiovascular surgery, gynecology, and maxillofacial surgery. 3D printing has proven to be a useful tool for the surgeon in many ways, both during the planning of surgery and during its execution [45,46,47].

Maxillofacial surgery is one of those branches of medicine in which 3D printing has broad applicability, both because of the presence of anatomical structures that are easily acquired by CT scans and because of the need for increasingly precise and personalized surgical procedures. The pathologies of greatest interest are mainly traumatic injuries, specifically orbital floor and medial fractures. In second place are oncologic diseases that require greater precision and preoperative preparation to which 3D printing would lend itself [48,49].

Regarding the applicability of this technology in the management of pathologies and disorders affecting the anatomical region of the orbit, this systematic review was able to derive useful information to understand how 3D printing can be applied. By analyzing the included studies, it is possible to see how this tool has several applications. In fact, it is possible either to directly print the implant to be used to reconstruct a fracture or to make 3D models of the fracture or anatomical region affected by the pathology to model on them the implant or titanium mesh to be implanted. In addition, 3D printing makes it possible to print surgical instruments and resection templates that allow the surgeon to perform precise osteotomies based on the anatomical and functional characteristics of the individual patient. Not to be forgotten is another essential function: surgical education. In fact, the creation of 3D models allows the surgeon to have a better visualization of the pathology, enables a better understanding of what surgical movements and procedures to adopt, and serves young students and physicians as an educational tool.

By analyzing the advantages and disadvantages of 3D printing, this tool goes a long way toward improving preoperative planning, surgical accuracy, and outcomes both functionally and cosmetically. It also allows for reducing operative time and the risk of complications and re-intervention. Unfortunately, the risk of complications and the need for re-intervention is still present, but at a lower rate than with conventional surgery. On the other hand, making a 3D model takes time, both for the virtual surgical planning phase and for printing the model. In addition, depending on the type of printer and the type of material used, costs can be high.

We must point out several limitations that were encountered during the writing of this review. The topic of this study is very specialized, and there is not a large body of randomized clinical trials in the literature. In fact, we had to include case series as well, keeping in mind, however, that this type of article may have inherent limitations in design that affect the risk of bias, and the interpretation of the results must be carried out with caution. The lack of a strong presence of clinical trials may be because 3D printing was considered from the beginning to be an excellent and useful tool for the surgeon, and this did not drive strong interest in conducting this type of study.

Looking toward the future, one technology that will revolutionize the world of 3D printing will be bioprinting, that is, the ability to print and recreate damaged tissues or even organs using 3D printers. This, in our opinion, must be the new research front to which we pay more attention and interest, driven especially by the need to replace organs and regenerate tissues [50,51].

## 5. Conclusions

3D printing has proven to be an outstanding tool in the management of orbit pathology. Comparing the advantages and disadvantages of such technology, the former far outweigh the latter. In fact, it allows for better preoperative visualization and preparation for surgery, a reduction in operative time, complications, and the risk of reoperation, and improved surgical precision and both functional and aesthetic outcomes. Unfortunately, most of the studies included in our review are case series due to the lack of randomized clinical trials in this field. To better understand the medical impact of 3D printing in the management of orbital pathologies, studies with more evidence are needed, although in practical reality, it is well known how this technology is giving great advantages in the surgical management of such pathologies.

## Figures and Tables

**Table 1:** Operation and characteristics of different types of 3D printers.

Type of Printer	Functioning	Features
Stereolithography (SLA)	It uses photopolymer resins that are solidified layer by layer using a UV laser.	They are printers that offer high precision and fine detail and are ideal for complex and highly accurate anatomical models.
Selective Laser Sintering (SLS)	It uses powders of materials such as nylon or metals that are melted layer by layer through a high-power laser. It can also use small particles of thermoplastics or ceramics that are melted.	They are suitable printers for creating strong and robust implants. They can be suitable for printing tissue implants, implants, and surgical guides.
Fused Material Deposition (FMD)	It uses thermoplastic material filaments that are extruded and deposited layer by layer.	They are the cheapest and most accessible printers but have lower accuracy than SLA and SLS printers.
Powder Bed Fusion (PBF)	It uses metal powders that are melted by a laser or electron beam.	They are ideal printers for manufacturing implants from titanium and other biocompatible metals.
Digital Light Polymerization (DLP)	It is similar to SLA, but instead of using a UV laser to solidify the resins, it uses a digital projector.	They are printers that can offer high speed and accurate details.
Multijet Modelling (MJM)	It uses photopolymers and uses a piezoelectric print head under ultraviolet exposure to solidify.	These printers, like SLS printers, also offer a wide range of medical applications including implants, surgical guides, and prosthetics.
Direct Metal Laser Sintering (DMLS)	It uses metal powders that are distributed layer by layer. These particles are then fused by a high-power laser.	They are printers that enable the creation of complex metal components with high precision.

**Table 2:** Materials used in 3D printing and relative printer.

Material Used	DMLS	FDM	SLS	SLA
Acrylonitrile	×	×		
Hydroxyapatite				×
Nylon			×	
Plaster			×	
PMMA			×	
Polyamide			×	×
Polybutadiene-styrene resin				×
Polycarbonate		×		
Polystyrene			×	
Resin (acrylic)			×	×
Titanium	×	×	×	

Legend: DMLS: Direct Metal Laser Sintering; FDM: Fused Deposition Modeling; SLS: Selective Laser Sintering; SLA: Stereolithography. The sign “×” indicates the type of printer that can use that specific material.

**Table 3:** PICOS framework.

Participant	Patients with diseases, oncological and otherwise, of the orbital region.
Interventions	Use of 3D printing for orbital disorders management.
Comparators	N/A
Outcomes	Accuracy, precision, complications, and functional and aesthetic outcomes
Study design	Clinical trials, randomized clinical trials, prospective and retrospective cohort studies, technical notes, and case series.

Legend: N/A: not applicable.

**Table 4:** Main features and implantability of the materials used in the included studies.

Material Used	Features	Implantability
Porous titanium	Excellent corrosion resistance and good biocompatibility. Due to its porous structure, it promotes bone integration. It has excellent mechanical strength and good integration capabilities with biological tissues, making it valuable in the biomedical field compared with other printing materials.	YES
Photopolymerized plastic	It can be shaped with precision. It is lightweight and durable. It is cured by the application of UV light. This material allows for high-precision printing. It is used extensively in rapid prototyping because of its ability to define fine details.	NO
Wax	It is a versatile and easily moldable material. Because of its properties, it is an ideal support material in 3D printing.	NO
Porous polyethylene	Good mechanical strength and biocompatibility. It is lightweight, flexible, and moisture-resistant. Its chemical resistance makes it useful in many fields and not only in the medical field.	YES
Polymethyl methacrylate (PMMA)	Durable yet easily workable material. It is used as a cement for implants and fixing dentures. It is a material that is not printed directly. In this case, 3D printing is used to create the substrates to model this material.	YES
Acrylonitrile butadiene styrene (ABS)	Lightweight but strong plastic material. Presents good mechanical properties. It is a rigid material and has good impact resistance.	NO
Plaster	Well moldable material.	NO
Polyetheretherketone (PEEK)	Excellent mechanical strength. Radiolucent. Good biocompatibility. It has high thermal and chemical resistance.	YES
Polylactic acid plastic filament	Biodegradable material used for temporary im-plants.	YES, only for temporary implants.
Polyamide	Good mechanical and chemical resistance. Its excellent wear resistance makes it ideal for applications requiring durability.	NO

**Table 5:** Data extracted from the included studies.

	Topic of the Study	Sample (n° Patients/Type of Orbital Disease)	3D Printing Applications	Type of Material Used/Printer	Results and Parameters Used
Bachelet et al., 2018 [33]	Patient-specific orbital implant evaluation (PSI) through the analysis of surgical timing and postoperative complications.	Total of 12 patients with complex bone fractures of the unilateral orbit (8 patients with diplopia, 12 with enophthalmos).	Direct printing of a patient-specific implant (PSI).	Porous titanium.	Average operative time of 71 min. In total, 17% of patients needed a second operation for mispositioning. The interventions were performed without the aid of positioning guides.
Kang et al., 2018 [34]	Assessing orbital wall reconstruction using 3D models through the analysis of the volumes of orbital tissue within the bony orbit.	Total of 11 patients: 6 with floor fractures and 5 with medial wall fractures. Bilateral blowout fractures, combined bone fractures.	Fabrication of a photo-polymerizable plastic press with wax support to mold compost plant from porous polyethylene with embedded titanium.	Photopolymerized plastic with wax support andPorous polyethylene with embedded titanium.	There is no statistically significant difference between the volume of contralateral orbital tissue and that of the affected orbit after reconstruction. The direct printing of custom titanium implants is much more expensive than the presented technique. Increased precision and reduced operative time.
Kim et al., 2018 [35]	PSI design for orbital floor and medial wall fractures.	In total, 28 patients with orbital floor and medial wall fractures.	Fabrication of original and mirrored rapid prototyping (RP) skull models using mirroring techniques during VSP for the intra-operative modeling of Medpor^®^ titanium implants to fit the pathology-affected orbit.	Plaster material	Evaluated clinical features (enophthalmos, visual acuity, diplopia) and orbital tissue volume by CT scans. Symmetry improvement of the orbital volume compared with the contralateral healthy orbit is recorded.
Martinez-Seijas et al., 2018 [36]	Creating an implantable prosthesis with 3D printing.	Five patients with cranial and/or orbital defects (tumor or traumatic etiology).	Printing of an injection molding system for implantable prosthesis.	Polymethyl methacrylate, PMMA	After the follow-up period, there were no postoperative complications to cause the removal of the prosthesis. It proves to be a cost-effective, reliable, and accurate method.
Pang et al., 2018 [37]	Evaluation use of 3D models for modeling porous polyethylene implants in orbit floor reconstruction.	Two patients with orbital floor fractures complicated with diplopia.	Fabrication of a porous polyethylene model of the orbit floor to model porous polyethylene sheets, Medpor^®^, on it.	Acrylonitrile butadiene styrenePorous polyethylene (Medpor^®^, Kalamazoo, Michigan, USA)	Average operative time: 67 min. Comparing the operative times of other surgeries, the operative time was reduced by 30 min. Improved precision and reduced operative time and duration of anesthesia.
Le Clerc et al., 2019 [38]	Postoperative tolerance assessment of titanium implants.	In total, 11 patients underwent maxillectomy with orbital floor resection (2 for benign tumors and 9 for malignant tumors).	Direct printing of titanium PSI for orbit floor reconstruction.	Titanium, PorousiTi^®^ (Materialise^®^, Leuven, Belgium	Parameters: need for re-intervention for infection or extrusion, functional characteristics, aesthetics, and quality of life. Only 18% of patients had implant extrusion. No cases of infection were reported. Good tolerance, high implant stability, reduced operative time, and satisfactory aesthetic results
Blumer et al., 2021 [39]	Evaluation of the use of titanium PSI for unilateral orbital fracture reconstruction.	In total, 34 patients with unilateral orbital fracture.	Fabrication of a 3D model of the fracture-affected area to model the titanium mesh during surgery.	Titanium mesh	To evaluate the outcome of the surgery, preoperative virtual reconstruction images were superimposed with postoperative 3D images, in addition to analyzing clinical data. Diplopia was recorded in 8.8% and enophthalmos in 11.8% after the follow-up period. Tool shown to be useful in improving surgical accuracy and reducing the operative time and long-term sequelae.
Yo et al., 2022 [40]	To compare the decompression effect at the optical channel by three different procedures through the study on 3D decompression models.	Nine patients with dysthyroid optic neuropathy.	Fabrication of 3D models to study the decompressive effect on the optic nerve channel of three different procedures: medial, balanced, and inferomedial.	Plaster	3D models prove to be useful for research and education, proving to be helpful in studying which decompression approach may be best for the individual and specific patient.
Zhao et al., 2023 [41]	Use of 3D printing for orbital reconstruction with iliac bone grafts.	Five patients with hemifacial microsomia.	Design of surgical guides, fixation plates, and titanium mesh for orbital and zygomatic reconstruction.	Titanium	Printing these materials allows for more precise, predictable, and effective interventions.
Cárdenas et al., 2024 [42]	Use of PSI in PEEK for complex orbit-cranial reconstruction.	In total, 15 patients (7 meningiomas, 5 benign tumor lesions, 2 malignant tumor lesions, and 1 trauma).	Fabrication of resection guides and polyetheretherketone (PEEK) implants.	Polyetheretherketone (PEEK)	The use of this technology demonstrates better results in terms of oncologic margin control, functional restoration, and aesthetic results.
Rama et al., 2023 [43]	Use of 3D models for orbit fracture education.	N/A	Making 3D models to educate trainees on orbit fractures.	White polylactic acid plastic filament with polyvinyl acetate media	It is shown how 3D-printed models can be useful for training trainees to improve their knowledge about pathologies of the orbit.
Lichtenstein et al., 2017 [44]	Use of 3D models for orbital surgical education.	N/A	Making 3D models to edicate trainees on pathologies of the orbit.	Polyamide	It is shown how 3D-printed models can be useful for training trainees to improve their knowledge about pathologies of the orbit

Legend: N/A: Not applicable.

**Table 6:** Advantages and disadvantages of 3D printing for orbital diseases management.

Advantages	Disadvantages
Better preoperative planning	Time requirements for VSP and 3D printing.
Precision of intervention	Cost and availability of materials and printers
Reduced operating time	Possibility of re-intervention
Better functional and aesthetic outcomes	
Reduction in postoperative complications and risks	
Better surgical education	
Reduced re-intervention rate	

## Data Availability

The data are available in a publicly accessible repository.
